# Favorable 10-year outcomes of osteochondral autologous transplantation for spontaneous osteonecrosis of the knee following subchondral insufficiency fracture with optimal alignment correction

**DOI:** 10.1186/s43019-025-00285-2

**Published:** 2025-08-12

**Authors:** Kohei Nishitani, Yasuaki Nakagawa, Masahiko Kobayashi, Shinichiro Nakamura, Shogo Mukai, Shinichi Kuriyama, Shuichi Matsuda

**Affiliations:** 1 Department of Orthopaedic Surgery, Graduate School of Medicine, Kyoto University, 54 Shogoin-Kawahara-Cho, Sakyo, Kyoto, 606-8507 Japan; 2 Department of Orthopaedic Surgery, Japan Baptist Medical Foundation, 47 Yamanomoto-Cho, Kitashirakawa, Sakyo, Kyoto, 606-8273 Japan; 3 Department of Orthopaedic Surgery, Kyoto Shimogamo Hospital, 17 Shimogamo-Higashimorigamae-Cho, Sakyo, Kyoto, 606-0866 Japan; 4 Department of Orthopaedic Surgery, National Hospital Organization Kyoto Medical Center, 1-1 Fukakusa-Mukaibata-Cho, Fushimi, Kyoto, 612-0861 Japan

**Keywords:** Osteonecrosis, Knee, Alignment, Cartilage, Repair

## Abstract

**Purpose:**

The long-term effect of osteochondral autologous transplantation (OAT) on spontaneous osteonecrosis of the knee (SONK) following subchondral insufficiency fracture remains unclear. This study aimed to evaluate the long-term survivorship and clinical outcomes of OAT for SONK, with a focus on factors associated with clinical success.

**Methods:**

Patients who underwent OAT for SONK between 1998 and 2009 were retrospectively reviewed. Survivorship was assessed using Kaplan–Meier analysis, with revision surgery as the endpoint. Clinical outcomes were evaluated using the International Knee Documentation Committee (IKDC) subjective score obtained preoperatively and at final follow-up. Clinical failure was defined as an IKDC score below the patient acceptable symptom state (PASS; 62.1 points). The association between final IKDC score and postoperative femorotibial angle (FTA) was analyzed using linear and quadratic regression.

**Results:**

A total of 33 OATs were included (mean age: 64.6 ± 8.0 years; mean lesion size: 3.9 ± 1.7 cm^2^). High tibial osteotomy was performed in 15 patients with FTA > 180°, and 24 patients were followed for ≥ 10 years (mean: 13.7 ± 3.4 years). One arthroplasty was performed at 14.2 years, yielding a 15-year survival rate of 88%. The IKDC score improved significantly (35.0 ± 12.6 to 70.6 ± 14.1, *p* < 0.001), with a clinical success rate of 79.2%. Quadratic regression showed optimal postoperative FTA between 163.1° and 178.3° for achieving PASS.

**Conclusions:**

OAT may provide favorable long-term survivorship and clinical outcomes in SONK, particularly when postoperative alignment is appropriately corrected.

## Introduction

Osteonecrosis of the knee is delineated into three entities: spontaneous osteonecrosis, secondary osteonecrosis, and post-arthroscopic osteonecrosis. Spontaneous osteonecrosis of the knee (SONK), which mainly occurs in medial femoral condyle, was recently reported as osteonecrosis subsequent to subchondral bone insufficiency fracture (SIFK); while secondary osteonecrosis is the osteonecrosis secondary to vascular insufficiency in the subcortical bone and bone marrow, represented by corticosteroid-induced osteonecrosis [1–3]. Although conservative treatment is the first choice of treatment, 47% of patients with SONK eventually require surgical intervention after 5 years of follow-up [1]. The presence of varus deformity (with a femorotibial angle [FTA] of 180° or more) is one of the well-known factors, which is significantly associated with poor prognosis of the conservative treatment of SONK [4].

There are many options in the surgical treatment strategies for SONK. Arthroplasties, such as total knee arthroplasty (TKA) and unicompartmental knee arthroplasty (UKA), provide promising pain relief for older and less active patients [5, 6]. For younger and more active patients, joint preservation surgeries, such as arthroscopic debridement, core decompression, osteochondral autograft transplantation (OAT), osteochondral allograft transplantation, and high tibial osteotomy (HTO), could be performed [7, 8]. Among them, OAT is one of the most common surgical treatments for cartilaginous and osteochondral disease, and good clinical results have been reported for focal cartilages lesion, osteochondritis dissecans, and various kind of osteonecrosis of the knee, ankle, elbow, and hip joint [3, 9–13]. Good short- and midterm results have been reported for SONK, but the long-term outcomes of OAT for SONK remain unknown [14–17].

Concomitant knee malalignment may impair the healing of the repaired cartilage by causing overload of the diseased compartment [8]. To decrease overload on the transplanted plug and accelerate healing of the repaired cartilage, realignment procedures are performed in cases of varus alignment. In a very recent article, for autologous chondrocyte implantation, slight valgus (2.5°) to varus alignment (4.5°) was recommended in 8.2 years of follow-up [18]. However, the degree of varus alignment required for alignment correction is unknown for OAT. This study firstly aimed to evaluate the long-term outcomes of OAT for SONK with and without HTO in terms of longevity and clinical scores. The secondary aim was to evaluate the factors that were associated with poor clinical outcomes. We hypothesized that OAT would demonstrate favorable long-term survivorship and clinical outcomes when postoperative alignment falls within an appropriate range.

## Methods

This retrospective case series was approved by the institutional review board of our hospital and conducted in accordance with the Declaration of Helsinki. Owing to the retrospective design, the requirement for written informed consent was waived, and an opt-out option was provided. Patients who underwent OAT for SONK of medial femoral condyle between 1998 and 2009 were enrolled in this study. Osteonecrosis was diagnosed using plain radiography and magnetic resonance imaging (MRI). Anteroposterior, lateral, and skyline plain radiographs were routinely taken. An anteroposterior long-leg radiograph was also taken to evaluate long leg alignment. The indications for an OAT to be undertaken were as follows: patients who had extensive osteochondral lesions (> 1 cm^2^) only in the medial femoral condyle with Koshino classification stage II or more, irresponsiveness to conservative treatment of more than 3 months, and unwillingness to undergo joint replacement surgery. Kellgren–Lawrence (KL) grade 4 osteoarthritis was not considered a contraindication if the patient strongly desired to return to a high level of activity; however, KL grade 3 or less was regarded as more suitable for this procedure. FTA, which is the lateral angle between the anatomic axes of the distal femur (the femoral mid-shaft 10 cm proximal to the center of the femoral notch—the center of the femoral notch) and the proximal tibia (the center of the tibial spines—the tibial mid-shaft 10 cm distal to the center of the tibial spines), was measured on an anteroposterior short knee radiograph [19]. If the FTA was 180° or more or if the weight-bearing line passed on the necrotic lesion in the long-leg radiograph, realignment HTO was also performed. Contraindications of OAT included the following: knees with inflammatory diseases, knees with ligament abnormalities, large exposure of subchondral bone in the ipsilateral medial tibial plateau, knees with lateral condyle lesions, and knees with severe patellofemoral lesions. Patients who followed up for at least 10 years after the procedures were included in the analyses.

### Surgery

All surgeries were performed under general anesthesia. Diagnostic arthroscopy was performed to confirm that the location and size of the lesions were adequate for OAT. The medial parapatellar approach was used and OAT was performed after removal of necrotic tissue, using the Osteochondral Autograft Transfer System (OATS; Arthrex, Naples, FL, USA), with the non-weight-bearing area of the medial and/or lateral trochlea of the ipsilateral femur as donor sites. Lateral closed or medial opening-wedge HTO was performed in patients who underwent concomitant HTO. The target alignment was FTA of 170°. The rehabilitation protocol was the same for those who underwent OAT and OAT + HTO. Weight-bearing was prohibited for 2–3 weeks, and full weight-bearing was allowed after 6–7 weeks postoperatively.

### Clinical evaluations

The range of motion of the knee was measured before surgery and at the final follow-up using a handheld goniometer. The degree of SONK was evaluated using the Koshino classification using anteroposterior plain radiographs. Preoperative, postoperative, and final FTAs were evaluated using anteroposterior plain radiography to assess the change in knee alignment, and the preoperative and final KL grades of the femorotibial and patellofemoral joints were individually evaluated to detect the occurrence or progression of osteoarthritis. The International Knee Documentation Committee (IKDC) subjective score and Japanese Orthopaedic Association score for knee osteoarthritis (JOA knee score) were used to evaluate the clinical outcomes before surgery and at the final follow-up.

### Historical controls

To compare outcomes of patients treated with OAT + HTO, a historical control cohort was extracted from previously published studies. Included cases were those treated with HTO for SONK or SIFK without OAT or other cartilage/chondrocyte transplantation procedures. Patient backgrounds and survivorship were compared, using revision surgery as the endpoint.

### Statistics

Two failures were defined: one was any revision surgery on the transplanted plugs, such as TKA, revision OAT, or autologous chondrocyte implantation, and the other was the IKDC subjective score below the patient acceptable symptom state (PASS). The PASS represents the minimum symptom level that distinguishes patients who consider themselves well from those who do not. In this study, we adopted a cutoff score of 62.1 points on the basis of a previously established PASS value for knee cartilage repair [20], Chahal et al. Survival was evaluated using the Kaplan–Meier method. Pre- and final clinical scores of the patients with more than 10 years follow-up were compared with Wilcoxon signed rank test. To investigate the cause of failure in the PASS definition, scatter plots between the final IKDC subjective score and parameters, which may be associated with clinical outcomes (age, lesion size, and alignment), were drawn, and linear or quadratic curve approximation was performed. The coefficient of determination (*R*^2^) and coefficient of maximum degree(s) in the equation were calculated with 95% confidence intervals (CIs). All statistical analyses were performed using the JMP Pro 15 (JMP Statistical Discovery, Cary, NC, USA). There were no missing data to be imputed. Data were shown as mean ± standard deviation or median with interquartile range, and *p*-values < 0.05 were considered significant.

A priori sample size calculation was performed using G*Power 3.1.9.6 (Heinrich-Heine-Universität Düsseldorf, Düsseldorf, Germany), with power = 0.80, alpha error = 0.05, and standard deviation = 15 to obtain a difference of more than minimum clinically important difference of IKDC subjective score [21], and *n* = 21 was required.

## Results

**Fig. 1:**
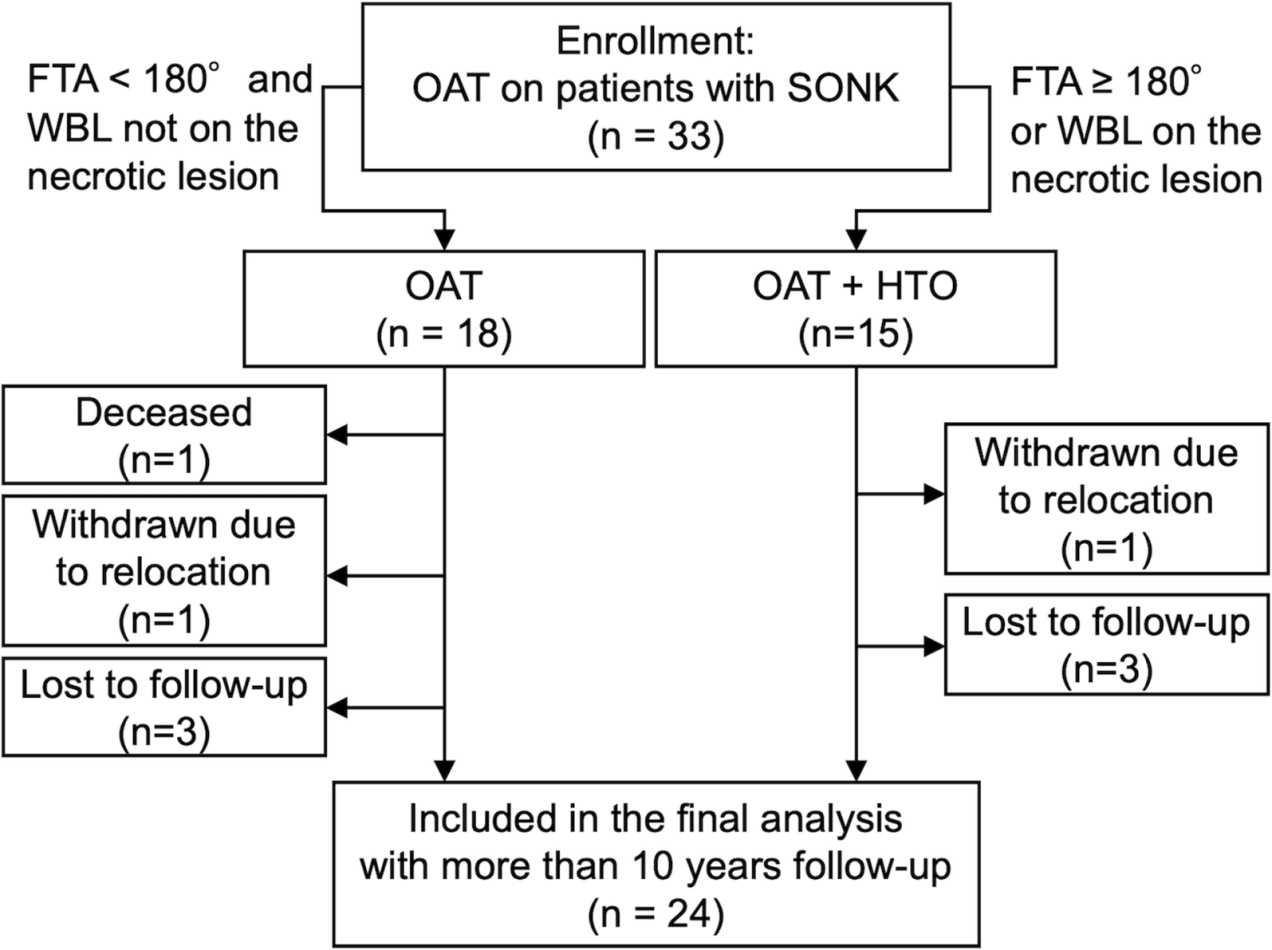
Flow diagram of this study. *SONK* spontaneous osteonecrosis of the knee, *FTA* femorotibial angle, *OAT* osteochondral autologous transplantation, *HTO* high tibial osteotomy

During the study period, 33 OAT were performed for SONK and included in this study (Fig. 1, Table 1). Concomitant HTO was performed in 15 patients (Table 1). A mean of 3.9 cm^2^ lesion in medial femoral condyle was treated with a median of three plugs (Table 2). For the OAT + HTO surgeries, the mean correction angle was 11.5 ± 1.7°, and the postoperative FTA was 170. ± 1.7°, which became more valgus compared with OAT-only patients (177.0 ± 1.8°). Among them, one patient who underwent OAT died 8.9 years after surgery; one patient who underwent OAT and one who underwent OAT + HTO were withdrawn due to relocation and referral to other hospitals within 10 years; and three patients in each surgical category were lost to follow-up (Fig. 1). Thus, 24 patients were included in the final follow-up period. Mean follow-up duration was 13.7 ± 3.4 (minimum: 10.1 and maximum: 20.8) years (Table 3).

**Table 1:** Preoperative demographics

Variables	Total (*n* = 33)	OAT (*n* = 18)	OAT + HTO (*n* = 15)
Age (years)	64.6 ± 8.0	66.3 ± 7.1	63.8 ± 8.8
Sex	27 female, 6 male	16 female, 2 male	11 female, 4 male
Operated side	18 left, 15 right	11 left, 7 right	7 left, 8 right
Preoperative FTA (°)	179.0 ± 3.1	176.9 ± 1.8	181.5 ± 2.3
Koshino classification	4 (3–4)	3 (2–3)	4 (3–4)
KL grade (FT)	2 (2–3)	2 (2–3)	2 (2–3)
KL grade (PF)	0 (0–1)	0 (0–1)	0 (0–1)
Knee extension (°)	−4.6 ± 8.1	−3.9 ± 9.5	−5.5 ± 6.1
Knee flexion (°)	135.0 ± 10.6	134.7 ± 11.2	135.3 ± 10.3

Data are presented as actual count, mean ± standard deviation, or median (interquartile range). *FTA* femorotibial angle, *KL* Kellgren–Lawrence, *FT* femorotibial, *PF* patellofemoral

**Table 2:** Details of surgeries

	Total (*n* = 33)	OAT (*n* = 18)	OAT + HTO (*n* = 15)
Lesion size (cm^2^)	3.9 ± 1.7	3.5 ± 1.4	4.1 ± 1.8
Plug number	3 (2–4)	3 (2–4)	3 (3–3)
Preoperative FTA (°)	179.0 ± 3.1	176.9 ± 1.8	181.5 ± 2.3
Correction angle (°)	–	–	11.5 ± 1.7
Postoperative FTA (°)	173.8 ± 3.9	177.0 ± 1.8	170.0 ± 1.7

Data are presented as mean ± standard deviation, or median (interquartile range). *OAT* osteochondral autologous transplantation, *HTO* high tibial osteotomy, *FTA* femorotibial angle, *KL* Kellgren–Lawrence, *FT* femorotibial, *PF* patellofemoral

**Table 3:** Demographics and outcomes at final follow-up

Variables	(*n* = 24)	OAT (*n* = 13)	OAT + HTO (*n* = 11)
Age (years)	78.0 ± 6.6	76.5 ± 5.6	79.8 ± 7.5
Sex	20 female, 4 male	12 female, 1 male	8 female, 3 male
Operated side	12 left, 12 right	7 left, 6 right	5 left, 6 right
Final FTA (°)	174.4 ± 6.7	179.5 ± 3.1	168.4 ± 4.6
KL grade FT	2 (2–3)	3 (2–3)	2 (2–3)
KL grade PF	1.5 (1–2)	2 (1–2)	1 (1–2)
Knee extension (°)	−2.3 ± 3.6	−1.9 ± 3.3	−2.7 ± 4.1
Knee flexion (°)	139.0 ± 10.2	140.0 ± 12.0	137.7 ± 7.6
Follow-up duration (years)	13.7 ± 3.4	12.5 ± 2.7	15.1 ± 3.6

Data are presented as actual count, mean ± standard deviation, or median (interquartile range). *FTA* femorotibial angle, *KL* Kellgren–Lawrence, *FT* femorotibial, *PF* patellofemoral

**Fig. 2:**
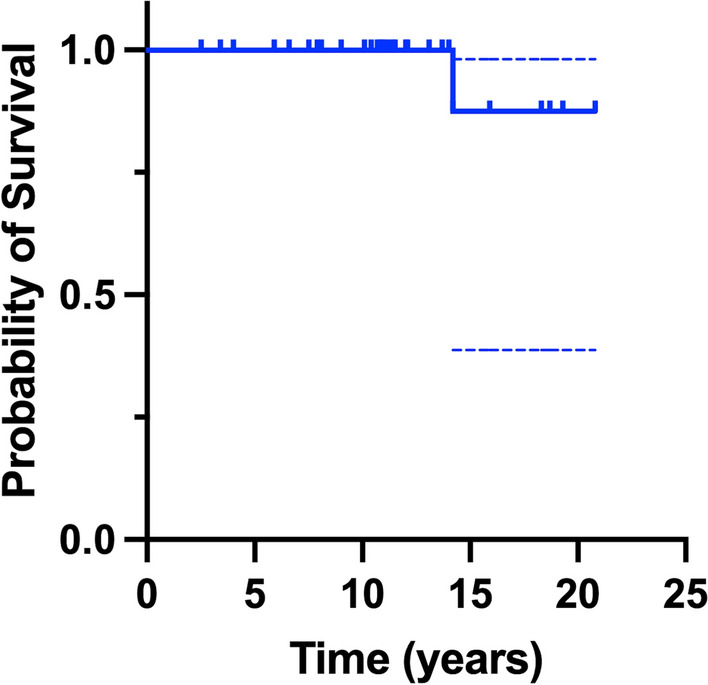
Kaplan–Meier analysis of any revision surgery for transplanted plugs. Dashed line indicates 95% confidential interval

**Fig. 3:**
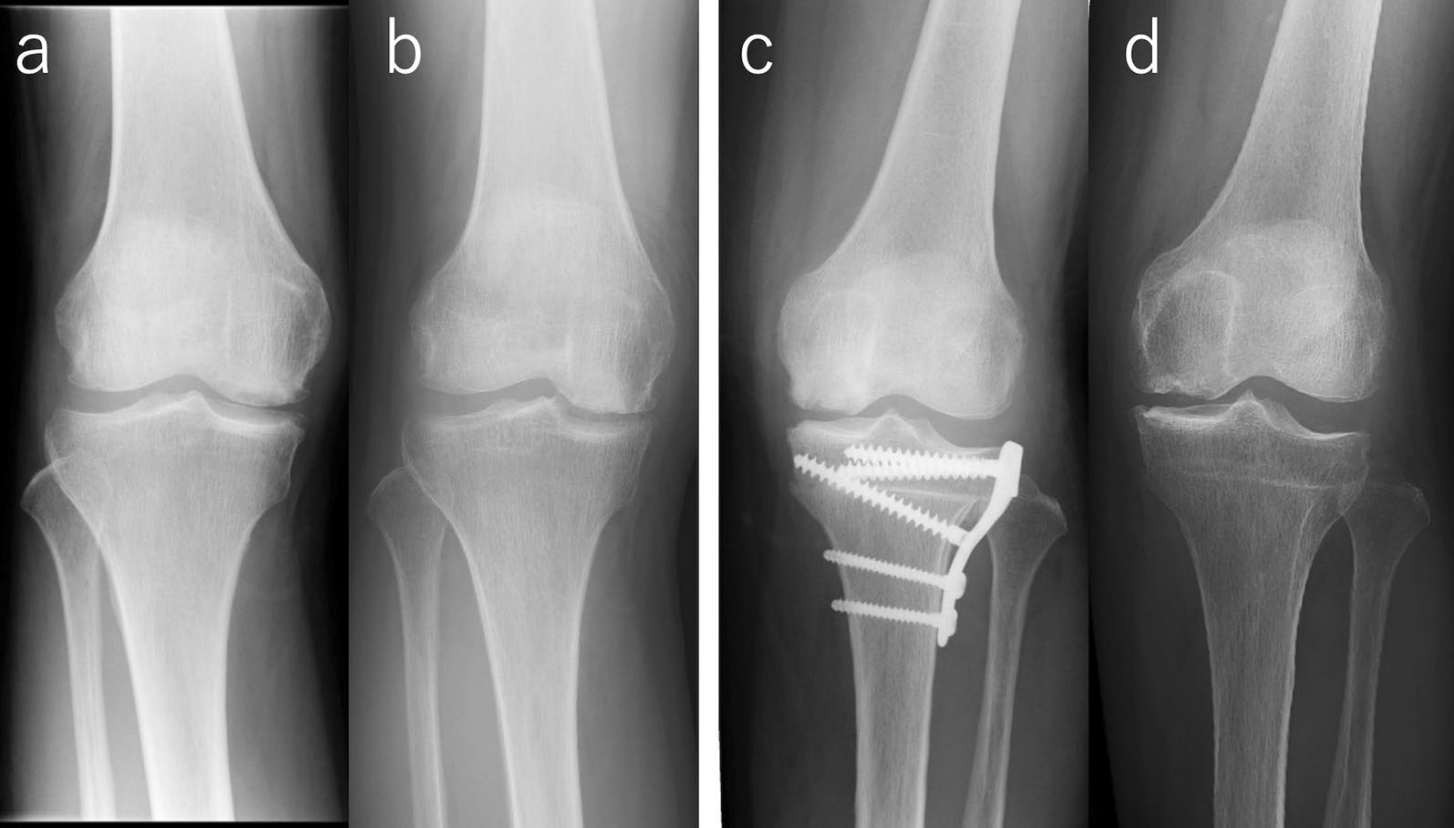
Postoperative (**a**, **c**) and final follow-up (**b**, **d**) radiographs of (**a**, **b**) a 53-year-old female patient with OAT whose FTA changed from 179.0° to 180.5° at 14.2 years after surgery, and who subsequently underwent revision TKA; and (**c**, **d**) a 63-year-old female patient with OAT + HTO whose FTA remained unchanged at 168.5° at 19.3 years after surgery

**Fig. 4:**
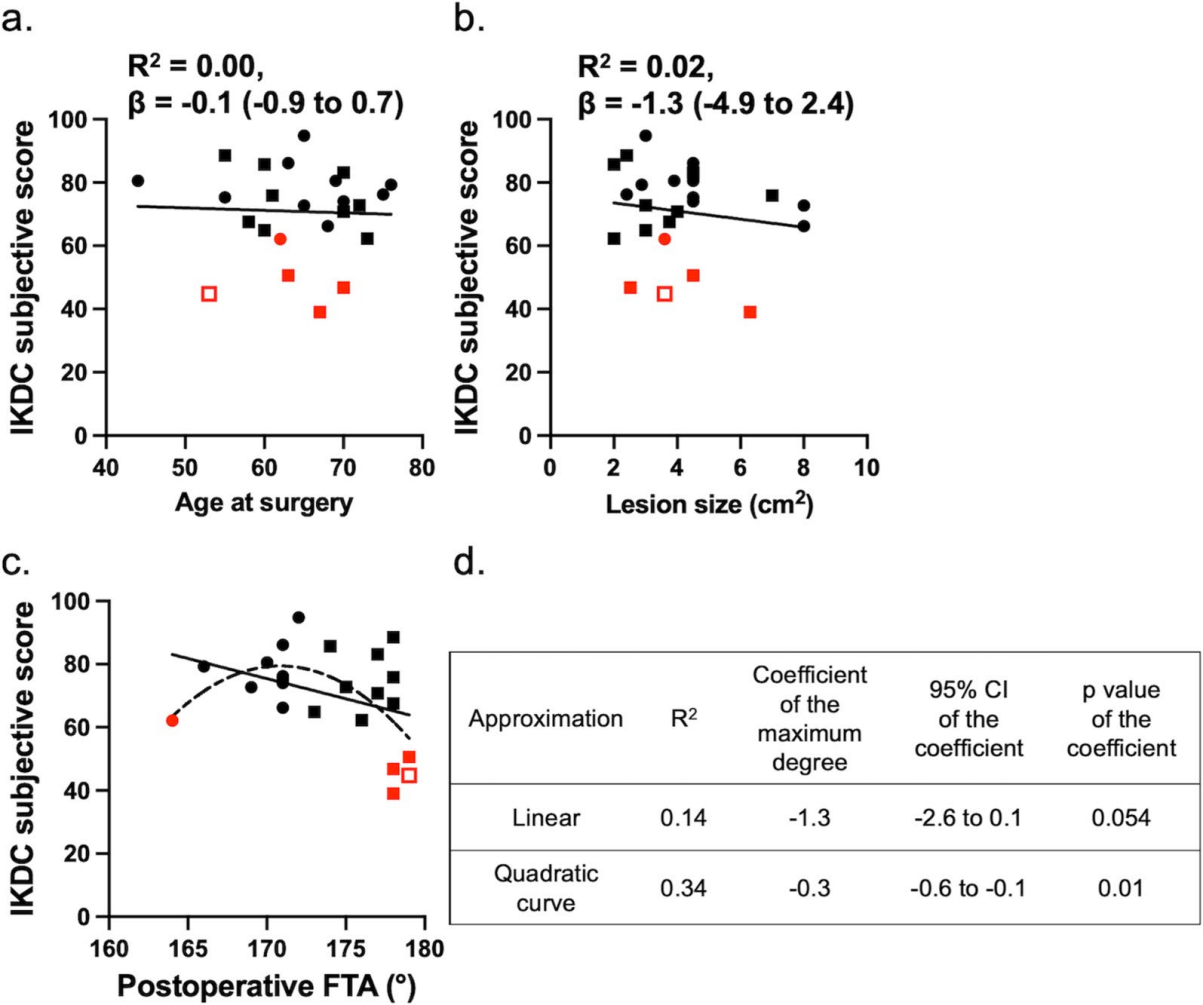
Association between the final International Knee Documentation Committee (IKDC) score and age at surgery (**a**), lesion size (**b**), and postoperative FTA (**c**). Lines and dashed line indicate linear and quadric curve regressions, respectively. Coefficient of determination (*R*^2^) and coefficient of maximum degrees in equations with 95% confidential intervals and *p*-values of the lines in (**c**) are shown in (**d**). Square is osteochondral autologous transplantation and circle is osteochondral autologous transplantation with concomitant high tibial osteotomy. Red color means patients with scores less than patient acceptable symptom state. Open red square is the revised patient

One revision TKA was performed 14.2 years after the primary surgery in a patient who underwent OAT without HTO (Fig. 2). This patient exhibited progression of osteoarthritis, from KL grade 2 at the time of the initial surgery to KL grade 3 before the revision, along with a slight increase in varus alignment from 179° to 180.5°. No other patient underwent revision surgery during the follow-up period, including those who dropped out within 10 years. Accordingly, the survival rates were 100% and 88% at 10 and 15 years, respectively, using revision surgery as the endpoint. However, because only 24 patients were observed beyond 10 years and just one revision event occurred, the survival estimate at 15 years should be interpreted with caution due to the wide confidence interval. Representative postoperative and final follow-up radiographs of patients who underwent OAT and OAT + HTO are shown in Fig. 3.The IKDC subjective and JOA knee scores significantly improved at the final follow-up (Table 4). Four patients with OAT and one patient with OAT + HTO showed IKDC scores less than the PASS at the final follow-up, resulting in a clinical score success rate of 79.2%. Age at surgery and lesion size showed no association with the final IKDC score (Fig. 4a, b). As shown in Fig. 4c, five patients with IKDC scores less than PASS had the lowest or highest postoperative FTA. Postoperative FTA showed better association with final IKDC score in quadratic curve approximation (*R*^2^ = 0.34) than in linear regression (*R*^2^ = 0.14). From the equation of the quadric regression, the best IKDC scores would be predicted when postoperative FTA was 170.9°. FTA values more than 163.1° and less than 178.3°, and more than 165.1 and less than 176.5°, were required to achieve the PASS and average IKDC score of this study, respectively.

**Table 4:** Preoperative and final clinical scores in patients who were followed-up more than 10 years (*n* = 24)

	Preoperative	Final	*p*-Value
IKDC subjective score	35.0 ± 12.6	70.6 ± 14.1	< 0.001
JOA knee score	59.3 ± 11.4	86.1 ± 12.0	< 0.001

Data are presented as mean ± standard deviation. *IKDC* International Knee Documentation Committee, *JOA* Japanese Orthopaedic Association

When OAT + HTO was compared with historical controls in which SONK was treated using HTO without OAT or other cartilage or chondrocyte transplantation procedures, all identifiable cases included concomitant marrow-stimulating techniques aimed at cartilage repair (Table 5). In four previous studies [15, 22–24], a total of 131 patients underwent HTO, and only one revision surgery was reported during mid-term follow-up. Patient age in the historical cohorts was generally comparable to that of in this study, and three of the four studies had a female-predominant population. The follow-up durations in those studies, reported as either mean or median values, ranged from 2.5 to 3.9 years, which were substantially shorter than that of our cohort.

**Table 5:** Comparison of OAT + HTO and historical HTO cohorts in SONK

	This study	Choi et al.* [22]	Chiu et al. [23]	Takeuchi et al. [21]	Kumagai et al. [15]
Number of patients	11	33	40	30	28
Treatment	HTO + OAT	HTO + MFX	HTO + MFX	HTO + BMS	HTO + BMS
Age	64.3 ± 7.7	58.7 ± 4.3	Median: 65.0 (IQR 60.0–70.0)	71.0 ± 5.6 years	69.7 ± 6.9
Sex	8 female, 3 male	13 female, 20 male	20 female, 10 male	28 female, 2 male	23 female, 5 male
Revision rate	0/11	0/33	1/40	0/30	0/28
Mean follow-up periods (years)	15.1 ± 3.6 (range 11.0–20.8)	3.4 ± 0.9	3.9 (IQR 2.9–5.6)	3.3 (range 2.0–5.2)	2.5 (range 2.0–3.4)

*The study by Choi et al. included SIFK cases. *HTO* high tibial osteotomy, *SONK* spontaneous osteonecrosis of the knee, *OAT* osteochondral autologous transplantation, *MFX* microfracture, *BMS* bone marrow stimulation, *IQR* interquartile range

## Discussion

The most important finding of this study was that OAT with or without HTO, depending on the preoperative knee alignment, provided good survivorship at minimum 10 years of follow-up. Addition of another definition of failure by PASS gave the success rate of 79.2% at the final follow-up (19 successes and 5 failures). Out of all the factors that were associated with final IKDC score, postoperative knee alignment was determined as the most significant.

This study showed the long-term survivorship and good clinical outcomes of OAT with or without HTO for SONK. Data on the long-term outcomes of surgical treatment for SONK are scarce. Among 52 patients with SONK, treated with various UKA implants from 1984 to 2004, the revision rate was 7.7% during the average follow-up of 10.9 ± 4.8 years [25]. In the Kaplan–Meier analysis, survival rates using revision as the endpoint were 93.1% and 90.6% at 10 and 15 years, respectively. Good recovery of Knee Society Score (knee score: 92 ± 12 and function score: 81 ± 19) and Western Ontario and McMaster Universities Osteoarthritis Index (7.7 ± 11.4) were reported, at an unsatisfactory 12.2%. Another report of 29 UKAs on spontaneous and secondary avascular necrosis with a mean follow-up of 21 years showed that survivorship of the components free of revision for any cause was 92% at 15 years [5]. Good clinical scores were also reported using the Knee Society Score as 89 points (range 68–100) for the knee score and 83 (range 66–96) for the functional score. In our study, the comparative survival rate (10 years: 100% and 15 years: 88%) was observed using revision surgery as the endpoint, and IKDC subjective score was significantly improved at final follow-up. As for the clinical outcomes for OAT with HTO, as a midterm follow-up, Kumagai et al. reported the results of OAT with HTO for SONK on 43 patients with large lesion size (> 4 cm^2^) with 100% of 5-year survival and Knee Society Score of 85.0 ± 10.4 in the knee score and 84.2 ± 12.4 in the function score [14]. Although the follow-up period was short, Tanaka et al. reported good recovery of the Lysholm score at 92.3 points after 2.3 years of follow-up after only OAT for SONK. In terms of other treatment used for SONK, such as arthroscopic debridement, bone grafting, and core decompression, only short-term results of 2–3 years are available [26, 27].

Alignment is a critical factor in cartilage degeneration and repair as it plays an important role in disease development and progression. In a study of osteochondral allograft transplants, persistent postoperative malalignment occurred more frequently in failed grafts and was a risk factor for graft failure (hazard ratio 6.55) [8]. A previous study of OAT for the medial compartment and varus malalignment resulted in worse clinical outcomes if the malalignment was not corrected, suggesting that correction is very important in protecting the resurfaced area [11]. In a MRI evaluation study of OAT for SONK, alignment correction with concomitant HTO provided better modified magnetic resonance observation of cartilage repair tissue score than OAT alone [28]. In this study, the final IKDC score was associated with postoperative knee alignment in the secondary regression rather than the linear alignment. This means that both residual varus malalignment and overcorrected valgus alignment would result in poor clinical outcomes. According to the equation of the secondary regression analysis, the best IKDC score would be predicted when postoperative FTA was 170.9°, and FTA would be more than 163.1 and less than 178.3°, hence achieving the PASS. Adding the four residual varus patients with FTA of 178° and 179° resulted in clinical failure; hence, more aggressive alignment correction was needed, although our application of concomitant HTO was FTA > 180°. Of course, alignment correction should be performed carefully to avoid an overcorrected valgus malalignment. Considering the average IKDC score of this study, postoperative FTA between 166° and 176° may be good to achieve.

Other factors that potentially affect the clinical outcomes after cartilage repair include patient age and cartilage lesion size [7, 8, 29]. Previous studies have suggested that age is one of the factors affecting the outcomes of any cartilage repair procedures [8, 30]. Recent studies have also shown that OAT can be effective in older adults [16, 31]. Consistent with these studies, age at the time of surgery (64.6 ± 8.0) was not associated with clinical outcomes. However, this result did not indicate that OAT can be performed in any age range because this study did not involve patients in the 80s age group. Large lesion size may affect clinical outcomes after cartilage repair. Several studies have been limited to lesion size of 4 cm [2] or less for OAT [30, 32]. In contrast, good OAT results have also been reported for lesions larger than 4 cm [2, 3, 14]. A previous study showed failure of bone marrow stimulation with a defect size over 4 cm^2^ but successful cartilage repair after OAT regardless of the lesion size [15]. For osteochondral lesions, better survival rates and bone union of autografts compared with allograft transplantation have been well recognized, and a live graft with osteoconductive, osteoinductive, and osteogenic properties may provide better healing with appropriate debridement [33].

This study had some limitations. First, this was a retrospective case series with a limited number of patients and without a control group, thus the evidence level was limited. There is a potential for selection bias, as this study only included patients who were considered suitable for OAT and who consented to undergo the procedure. Patients who opted for alternative treatments such as UKA or TKA, as well as those who had persistent symptoms but declined surgery, were not included. As a result, the study population may be biased toward individuals with higher motivation for joint preservation or relatively favorable joint conditions. Second, in patients who underwent concomitant HTO, it remains unclear which procedure contributed more to the clinical outcomes. Isolated HTO might also be effective for the treatment of SONK, but the superiority of OAT + HTO over HTO alone could not be assessed within our cohort. To address this limitation, we included a historical control group in which patients underwent HTO without cartilage or chondrocyte transplantation procedures. However, even in these historical controls, marrow-stimulating techniques were performed, thus the efficacy of HTO alone for SONK could not be determined. Third, raw images, such as radiographs and MRI, were not available in all cases because films for the 1990s and the early 2000s were already thrown away due to the completion of the legal storage periods. Knee alignment with FTA was available from the database, but whole-leg alignments, such as the hip–knee–ankle angle, could not be measured. Moreover, recent studies suggest that SIFK may lead to SONK [34], and meniscal status, such as meniscal protrusion or posterior root tear of the medial meniscus, is an important cause of SIFK [1]. As the etiology, meniscal tears occur in 50–100% of patients with SONK, and the size of the medial meniscal extrusion was found to be related to the stage and volume of the SONK lesions [35]. It was ideal to investigate preoperative meniscal condition, but this was impossible due to unavailability of preoperative MRI films in several cases. Fourth, there may be measurement and interpretation biases because range of motion analysis, radiographic measurements, and JOA score acquisition were not blinded. Lastly, although the long-term joint survival rate observed in this study appears favorable, the retrospective design without a control group limits the strength of the evidence. Higher-level studies, such as prospective cohort studies or randomized controlled trials, are warranted to confirm the efficacy of OAT compared with alternative treatment options.

In conclusion, this retrospective cohort study suggests that OAT may be effective in improving the longevity and clinical outcomes of patients with SONK. Postoperative knee alignment was associated with clinical outcomes, and both residual varus alignment and overcorrection may yield limited clinical outcomes.

## Data Availability

The data that support the findings of this study are available from the corresponding author upon reasonable request.
